# Ubiquitin-Specific Protease 3 Deubiquitinates and Stabilizes Oct4 Protein in Human Embryonic Stem Cells

**DOI:** 10.3390/ijms22115584

**Published:** 2021-05-25

**Authors:** Byung-Ho Rhie, Ainsley Mike Antao, Janardhan Keshav Karapurkar, Min-Seong Kim, Won-Jun Jo, Suresh Ramakrishna, Kye-Seong Kim

**Affiliations:** 1 Graduate School of Biomedical Science and Engineering, Hanyang University, Seoul 04763, Korea; aaa0712@hanyang.ac.kr (B.-H.R.); ainsleyantao@hmail.hanyang.ac.kr (A.M.A.); janardhan25@hmail.hanyang.ac.kr (J.K.K.); roseday@hanyang.ac.kr (M.-S.K.); cwj960@hanyang.ac.kr (W.-J.J.); 2 College of Medicine, Hanyang University, Seoul 04763, Korea

**Keywords:** CRISPR/Cas9, embryonic carcinoma cells, gene knockout, post-translational modifications, 26S proteasome

## Abstract

Oct4 is an important mammalian POU family transcription factor expressed by early human embryonic stem cells (hESCs). The precise level of Oct4 governs the pluripotency and fate determination of hESCs. Several post-translational modifications (PTMs) of Oct4 including phosphorylation, ubiquitination, and SUMOylation have been reported to regulate its critical functions in hESCs. Ubiquitination and deubiquitination of Oct4 should be well balanced to maintain the pluripotency of hESCs. The protein turnover of Oct4 is regulated by several E3 ligases through ubiquitin-mediated degradation. However, reversal of ubiquitination by deubiquitinating enzymes (DUBs) has not been reported for Oct4. In this study, we generated a ubiquitin-specific protease 3 (USP3) gene knockout using the CRISPR/Cas9 system and demonstrated that USP3 acts as a protein stabilizer of Oct4 by deubiquitinating Oct4. USP3 interacts with endogenous Oct4 and co-localizes in the nucleus of hESCs. The depletion of USP3 leads to a decrease in Oct4 protein level and loss of pluripotent morphology in hESCs. Thus, our results show that USP3 plays an important role in controlling optimum protein level of Oct4 to retain pluripotency of hESCs.

## 1. Introduction

Embryonic stem cells (ESCs) are derived from the inner cell mass (ICM) of mammalian blastocysts and are characterized by their ability to differentiate into multiple cell types and undergo self-renewal [1]. ESC self-renewal and differentiation ability are mainly regulated by a network of transcriptional factors (1). Indeed, turnover of transcriptional factors such as octamer-binding factor 4 (Oct4), SRY box-containing factor 2 (Sox2), Krüppel-like factor 4 (Klf4), c-Myc, Nanog, Lin28, and Sall4 are mainly responsible for determination of the cell fate of ESCs [2,3,4,5,6].

Maintenance of pluripotency in human ESCs (hESCs) is a balance between expression of a key set of proteins comprising Oct4, Sox2, and Nanog [7,8]. Expression of Oct4 is high during the early embryonic stages and gradually declines during differentiation of hESCs, suggesting that Oct4 is a critical transcription factor in cell fate determination [9]. Moreover, the expression level of Oct4 is a major determinant of maintenance of the pluripotent status of hESCs [9,10].

Post-translational modifications (PTMs) of transcription factors, including Oct4, are fundamental regulators of cellular functions. Oct4 function is regulated by several PTMs including phosphorylation, ubiquitination, SUMOylation, and glycosylation [11,12,13,14,15]. Among several PTMs of Oct4, ubiquitination is a major regulator of Oct4 protein turnover [2,16]. The ubiquitination pathway is controlled by the ubiquitin proteasome system (UPS) that covalently ligates the small molecule ubiquitin (Ub) to specific sites on substrate proteins [17]. Several E3 ligases that regulate protein turnover of Oct4 have been reported, including the WW domain-containing E3 ubiquitin protein ligase 2 (WWP2) and Itch, which regulates Oct4 via K63-Ub linkages [12,18,19]. DPF2 or ubi-d4/requiem (REQU) regulates Oct4 via K48-Ub linkage, and CHIP is a ubiquitin ligase (E3 ligase) that regulates Oct4 in breast cancer stem cells [20,21]. Reversal of the ubiquitination process is performed by deubiquitinating enzymes (DUBs) that balance the action of E3 ligases to regulate key functions as well as the stability and localization of target proteins [22]. Although extensive research has been performed to elucidate the role of Oct4 ubiquitination in the maintenance of pluripotency in stem cells, little is known about the DUBs that regulate Oct4 protein level in hESCs. However, a recent study by Wei et al. reported that the transcription factor Bach1 interacts with and facilitates Nanog, Sox2, and Oct4 deubiquitination by recruiting USP7. Bach1-mediated recruitment of USP7 helps maintain the identity and self-renewal of hESCs while the loss of Bach1 affects the differentiation fates of these cells [23]. This report therefore highlights the importance of DUBs in regulating stem cell fate determination and differentiation by regulating the stability of key transcriptional factors in hESCs.

We recently demonstrated that USP3 interacts with and deubiquitinates the cell division cycle 25A (Cdc25A) protein, which regulates proper progression of the cell cycle [24]. Cdc25 proteins are responsible for activating CDK complexes during the cell cycle, and Cdc25A plays a role in regulating G1/S cell cycle progression [24]. Cdc25A is also known to regulate the cell cycle in stem cells [25,26,27]. Interestingly, gene ontology (GO) analysis identified a positive correlation between Oct4 and various cell cycle genes, including *Cdc25A* [28]. Previous studies have demonstrated that, in addition to Cdc25A, USP3 is a regulator of stemness associated-genes such as KLF5 and SUZ12 [29,30].

Thus, we hypothesized that USP3 has an important role in regulating the protein level of key transcriptional factors in hESCs. In this study, we performed a loss-of-function study of *USP3* in ESCs utilizing the CRISPR/Cas9 system. We demonstrate that USP3 interacts with and deubiquitinates endogenous Oct4 in hESCs. The loss of USP3 significantly destabilizes the protein level of Oct4 and affects normal morphology of hESCs.

## 2. Results

### 2.1. Generation of Single-Cell-Derived USP3 Gene Knockout Clones in Human Embryonic Carcinoma Stem Cells

**Figure 1:**
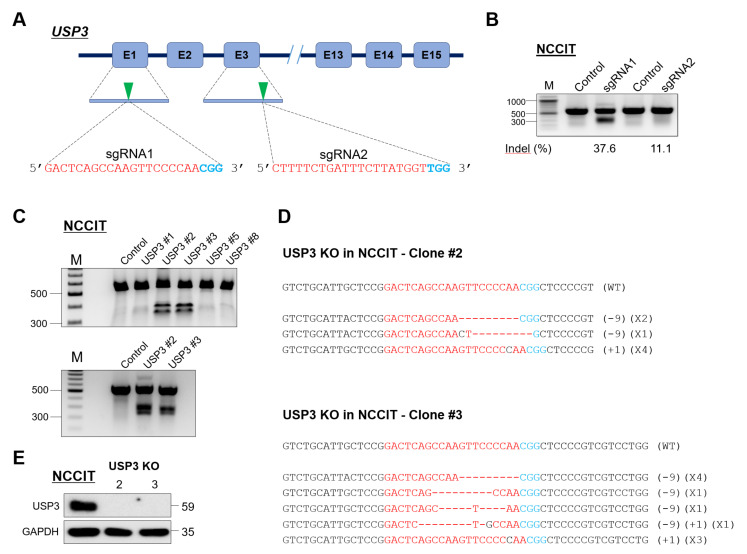
Generation of *USP3* knockout in NCCIT cells. (**A**) Schematic of RNA-guided engineered nuclease targeting of the human *USP3* gene using sgRNA1 and sgRNA2, which were designed to target sequences in exon 1 and exon 3, respectively. sgRNA target sequences are represented in red, and PAM sequences in blue. (**B**) T7E1 assays were performed in NCCIT cells to determine the cleavage efficiency of sgRNA1 and sgRNA2 by transfecting along with Cas9 plasmid. Samples were resolved in 2% agarose gel. The cleaved band intensity obtained from T7E1 assay were measured (indel %) using ImageJ software. Un-transfected NCCIT cells were used as control cells. A marker is shown for size reference. (**C**) USP3 knockout single-cell colonies were screened using the T7E1 assay (upper panel). The USP3 KO-positive clones, i.e., USP3 KO#2 and #3 were reconfirmed by T7E1 assay (lower panel). (**D**) USP3 gene-disrupted sequences obtained from Sanger sequencing, i.e., USP3 KO#2 (upper panel) and USP3 KO#3 (lower panel). The sgRNA recognition site is indicated in red, and the protospacer adjacent motif (PAM) is indicated in blue. Dashes indicate deleted bases, while inserted bases are represented in black. The number of deleted and inserted bases are mentioned in the parentheses; the numbers of occurrences of the indicated sequences are shown in parentheses (for example, X1 and X2 indicate the number of each clone sequenced). (**E**) USP3 knockout efficiency in NCCIT cells was checked by Western blot analysis for USP3 KO clones #2 and #3 using the USP3-specific antibody. GAPDH was used as loading control.

To elucidate the role of USP3 in embryonic stem cells, we generated single-cell-derived USP3 knockout clones in a human embryonic carcinoma cell line (NCCIT). NCCIT cells have gene expression profiles similar to those of embryonic stem cells [31] and were selected to investigate the effect of USP3 on the pluripotency of stem cells. To this end, we designed two sets of sgRNAs targeting *USP3*, namely, sgRNA1 and sgRNA2, that specifically target early exon 1 and exon 3, respectively, as illustrated in Figure 1A. Gene disruption efficiencies of the two sgRNAs were validated by the indel percentage observed by T7E1 assay. sgRNA1 showed a higher indel percentage then sgRNA2, so we chose sgRNA1 to generate single-cell-derived USP3 knockout (USP3 KO) clones (Figure 1B). NCCIT cells transfected with plasmids encoding Cas9 and sgRNA1 were subjected to single-cell dilution followed by seeding into 96-well plates. Individual single-cell-derived clones that showed *USP3* gene disruption were selected using the T7E1 assay (Figure 1C). T7E1-positive USP3 KO clones #2 and #3 were Sanger sequenced to confirm *USP3* gene disruption (Figure 1D). USP3 KO clones #2 and #3 were analyzed by Western blot analysis using endogenous USP3 antibody, and complete loss of USP3 protein expression in both clones were confirmed (Figure 1E).

### 2.2. USP3 Regulated Oct4 Protein Stability and Half-Life

**Figure 2:**
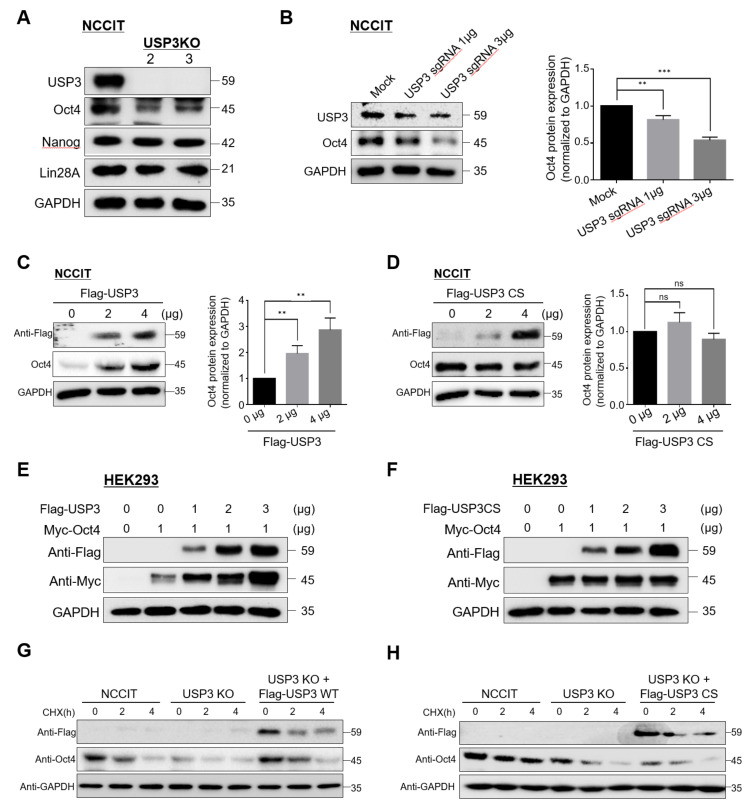
USP3 regulated Oct4 protein stability. (**A**) Protein expression of stem cell transcription factors upon deletion of *USP3* in USP3 KO NCCIT clones #2 and #3 were detected by Western blot analysis using the indicated antibodies. GAPDH was used as a loading control. (**B**) NCCIT cells were transfected with constant amount of Cas9 along with increasing concentrations of sgRNA targeting *USP3* (1:1 and 1:3 ratios, respectively). Depletion of USP3 and its effect on the endogenous expression of Oct4 protein was detected by Western blot. The band intensities of Oct4 proteins were estimated using ImageJ software and were graphically represented after normalization with GAPDH. Data were presented as the means ± SDs of three independent experiments (*n* = 3). Statistical analysis was conducted by Student’s *t*-test (** *p <* 0.01, *** *p* < 0.001). (**C**) NCCIT cells were transfected with increasing concentrations of wild-type Flag-USP3 (0, 2, 4 μg), and the endogenous expression of Oct4 was analyzed by Western blotting. The band intensity of Oct4 proteins were estimated using ImageJ software and graphically represented after normalization with GAPDH. Data were presented as the means ± SDs of three independent experiments (*n* = 3). Statistical analysis was conducted by Student’s *t*-test (** *p <* 0.01). (**D**) NCCIT cells were transfected with increasing concentrations of catalytic mutant Flag-USP3CS (0, 2, 4 μg), and the endogenous expression of Oct4 was analyzed by Western blotting. The band intensities of Oct4 proteins were estimated using ImageJ software and graphically represented after normalization with GAPDH. Data were presented as the means ± SDs of three independent experiments (*n* = 3). Statistical analysis was conducted by Student’s *t*-test (ns = not significant). (**E**) HEK293 cells were transfected with a constant amount of Myc-Oct4 (1 μg) along with increasing concentrations of Flag-USP3 (0, 1, 2, 3 μg), and the exogenous expression of Myc-Oct4 was analyzed by Western blotting. (**F**) HEK293 cells were transfected with a constant amount of Myc-Oct4 (1 μg) along with increasing concentrations of Flag-USP3CS (0, 1, 2, 3 μg), and the exogenous expression of Myc-Oct4 was analyzed by Western blotting. (**G**,**H**) The half-life of Oct4 was measured in USP3-expressing NCCIT cells, *USP3*-deleted NCCIT cells, and *USP3-*deleted NCCIT cells transfected with (**G**) Flag-USP3 or (**H**) Flag-USP3CS upon the treatment of 150 μg/mL protein synthesis inhibitor cycloheximide (CHX). Cells were harvested at different time points (0, 2, and 4 h) and analyzed by Western blotting.

Loss of USP3 in NCCIT cells resulted in a significant decrease in the protein level of Oct4, a master regulator of ESC pluripotency (Figure 2A). However, the protein expression levels of other pluripotent transcriptional factors such as Nanog and Lin28A were not significantly altered (Figure 2A). This suggested that USP3 might stabilize Oct4 protein level in NCCIT cells.

To investigate the stabilization effect of USP3 on Oct4, we dose-dependently increased the ratio of sgRNA targeting *USP3* while maintaining a constant ratio of Cas9 (Cas9: sgRNA; 1:1, and 1:3). A gradual decrease in USP3 protein expression due to an increase in sgRNA ratio targeting the *USP3* gene resulted in a dose-dependent decrease in Oct4 protein level in NCCIT cells (Figure 2B). In addition, when NCCIT cells were transfected with increasing concentrations of USP3, a dose-dependent increase in endogenous Oct4 expression was observed (Figure 2C). However, when NCCIT cells were transfected with increasing concentrations of *USP3* catalytic mutant USP3C168S (USP3CS), no increase in expression of endogenous Oct4 was observed (Figure 2D). Similarly, in HEK293 cells, we observed protein stabilization of ectopically expressed Myc-Oct4 as the concentration of Flag-USP3 increased (Figure 2E), but expression of the catalytic mutant Flag-USP3CS had no such stabilization effect (Figure 2F). Furthermore, treatment with a protein synthesis blocker-cyclohexamide (CHX) significantly reduced the half-life of Oct4 in *USP3*-depleted NCCIT cells, which could be rescued by the ectopic expression of USP3 (Figure 2G) but not by the catalytic mutant USP3CS (Figure 2H), indicating that USP3 acts as a protein stabilizer of Oct4.

### 2.3. USP3 Interacted with Oct4

**Figure 3:**
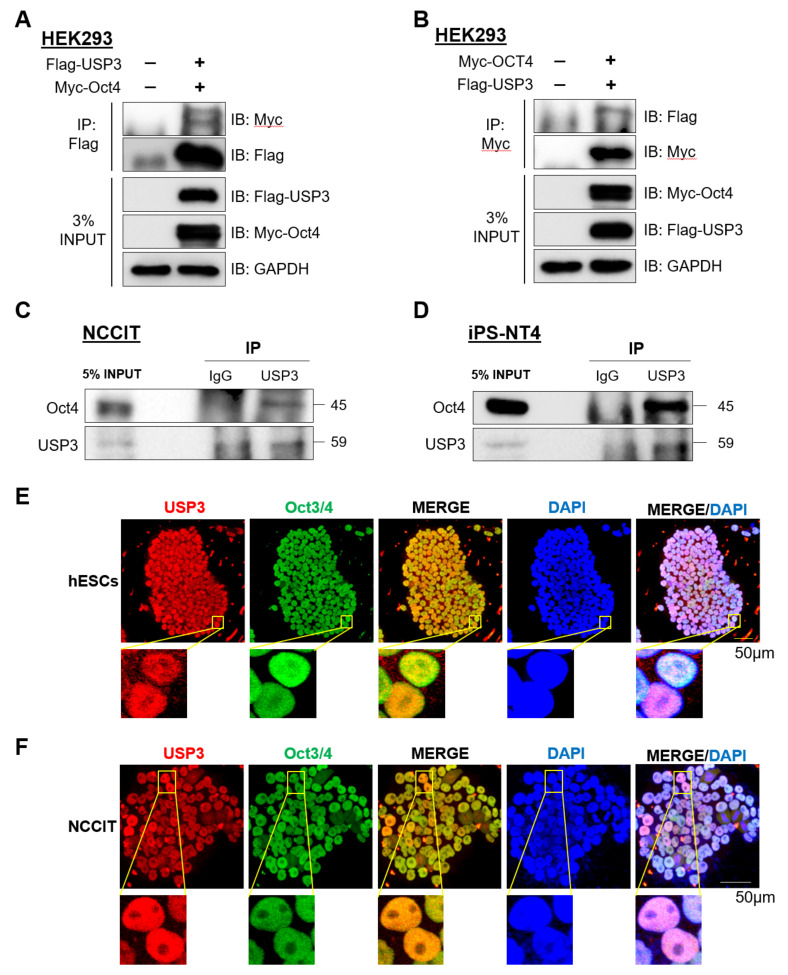
USP3 interacted and co-localized with Oct4. Exogenous immunoprecipitation (IP) was performed by co-transfecting Flag-USP3 and Myc-Oct4 into HEK293 cells. (**A**) IP was performed using anti-Flag antibody and immunoblotted using anti-Myc antibody. GAPDH was used as a loading control. (**B**) IP was performed using anti-Myc antibody, immunoblotted using anti-Flag antibody. GAPDH was used as a loading control. (**C**) Endogenous interaction between USP3 and Oct4 was demonstrated in NCCIT cells by performing IP using anti-USP3 antibody and immunoblotted with anti-Oct4 antibody. GAPDH was used as a loading control. (**D**) Endogenous interaction between USP3 and Oct4 in human iPSCs was demonstrated by performing IP with anti-USP3 antibody and immunoblotted with anti-Oct4 antibody. GAPDH was used as a loading control. (**E**) Co-localization analysis between endogenous USP3 and Oct4 was performed in hESCs by immunostaining with anti-USP3 and Oct4-specific antibodies. DAPI was used to stain the nucleus. (Scale bar: 50 μm). Inset represents threefold magnified images. (**F**) Co-localization analysis between endogenous USP3 and Oct4 was performed in NCCIT by immunostaining with anti-USP3 and Oct4-specific antibodies. DAPI was used to stain the nucleus. (scale bar: 50 μm). Inset represents threefold magnified images.

To determine the biological role of USP3 in embryonic stem cells, we first analyzed the physical association between USP3 and Oct4 in vivo. For this purpose, we performed immunoprecipitation (IP) experiments to investigate the interaction between USP3 and Oct4 at the exogenous level. We co-transfected Flag-USP3 and Myc-Oct4 into HEK293 cells and performed IP with Flag or Myc antibodies, followed by Western blot with reciprocal antibodies. Flag-USP3 co-precipitated Myc-Oct4 (Figure 3A), and similarly Myc-Oct4 co-precipitated Flag-USP3 (Figure 3B). Further, we performed endogenous IP experiments in NCCIT cells and human-induced pluripotent stem cells (hiPSCs). IP using endogenous USP3 antibody resulted in precipitation of endogenous Oct4 in NCCIT cells (Figure 3C) as well as in hiPSCs (Figure 3D), indicating that USP3 interacts stably with endogenous Oct4 in stem cells.

Next, we examined the cellular localization of USP3 and Oct4 in hESCs. Immuno-localization using specific antibodies in hESCs showed that USP3 and Oct4 are present in the nucleus, consistent with previous reports [24,32] (Figure 3E). As the nucleus accounts for a large part of the embryonic stem cell volume, we re-confirmed co-localization of USP3 with Oct4 in NCCIT cells. USP3 and Oct4 were expressed predominantly in the nucleus (Figure 3F). These data demonstrate that USP3 and Oct4 co-localize in the nucleus of both hESCs and NCCIT.

### 2.4. Deubiquitination of Oct4 by USP3

**Figure 4:**
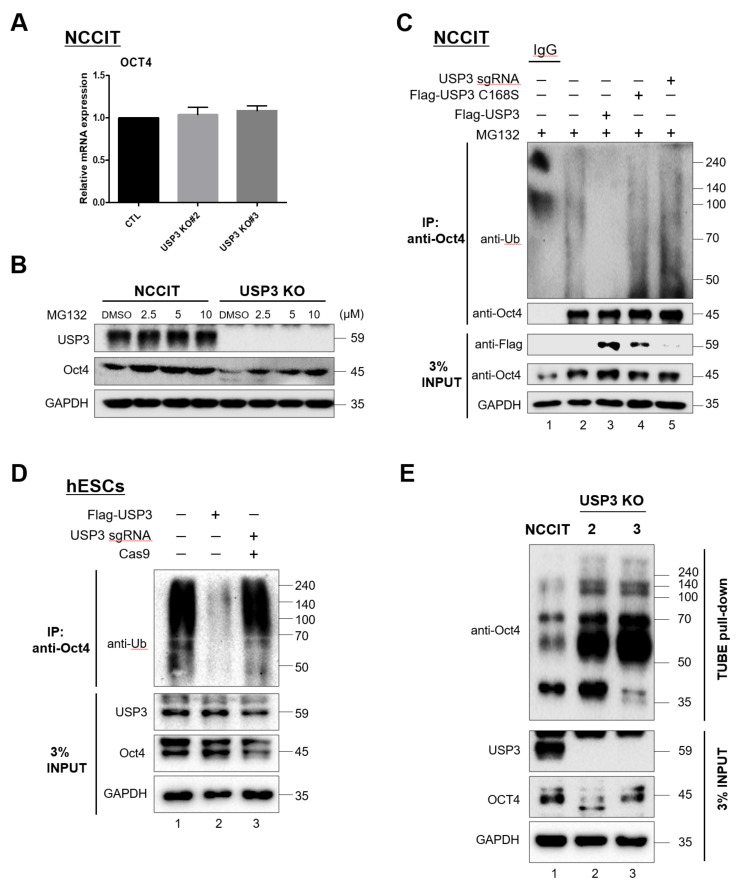
Deubiquitination of Oct4 by USP3. (**A**) The effect of *USP3* gene knockout on the mRNA expression of *Oct4* was analyzed by qRT-PCR analysis with specific primers. Representation of relative mRNA expression levels of *Oct4* after normalization with *GAPDH*. (**B**) USP3-expressing and USP3-depleted NCCIT cells were treated with the proteasome inhibitor MG132 (DMSO as control, 2.5, 5, or 10 μM MG132) for 6 h at the indicated concentrations and analyzed by immunoblotting against the indicated antibodies. GAPDH was used as a loading control. (**C**) For endogenous Oct4 deubiquitination assay in NCCIT, Flag-USP3, Flag-USP3CS, and sgRNA targeting *USP3* were transfected into NCCIT cells as indicated. Oct4 deubiquitination was confirmed by IP with Oct4-specific antibody and immunoblotted with the ubiquitin-specific antibody. (**D**) For endogenous Oct4 deubiquitination assay in hESCs, Flag-USP3 and *USP3*-targeting sgRNA were transfected into hESCs as indicated. Oct4 deubiquitination was confirmed by IP with Oct4-specific antibody and immunoblotted with ubiquitin-specific antibody. (**E**) Tandem ubiquitin binding entities (TUBEs) assay for the ubiquitination of Oct4 proteins were performed in USP3-expressing and USP3-depleted NCCIT cells treated with 10 μM MG132 for 6 h. Cell lysates were immunoprecipitated with agarose–TUBE beads and immunoblotted with Oct4-specific antibodies.

To investigate the mechanism of Oct4 stabilization by USP3 in stem cells, we analyzed mRNA expression patterns of *Oct4* in *USP3*-depleted NCCIT cells. Knockout of *USP3* in NCCIT did not alter the mRNA expression of *Oct4*, indicating that USP3 regulates Oct4 at the post-translational level but not at the transcriptional level (Figure 4A). Our observations were further confirmed by treating a proteasome inhibitor-MG132 that could rescue the expression of Oct4 proteins in USP3 depleted NCCIT cells (Figure 4B).

Oct4 was previously reported to undergo polyubiquitination via the 26S proteasomal pathway [19]. On the basis of the stabilizing function of USP3 on Oct4, we speculated that USP3 functions as a deubiquitinase regulating Oct4 protein turnover. To this end, we examined the polyubiquitination of Oct4 in the presence of USP3, the catalytic mutant USP3CS, or sgRNA targeting *USP3* in NCCIT and hESCs. Overexpression of USP3 exhibited deubiquitinating activity, which led to a decrease in the endogenous Oct4 polyubiquitinated smear in NCCIT (Figure 4C, lane 3) and in hESCs (Figure 4D, lane 2), but deubiquitinating activity was not observed in the presence of USP3CS (Figure 4C, lane 4) or upon knockdown of *USP3* (Figure 4C, lane 5; Figure 4D, lane 3). Furthermore, we validated the endogenous ubiquitination of Oct4 by performing a TUBE assay in the presence or absence of USP3 in NCCIT cells. The depletion of USP3 leads to an increase in the accumulation of ubiquitination of endogenous Oct4 proteins (Figure 4 E, lane 2 and 3 vs. lane 1).

Overall, our results indicate that USP3 stabilizes Oct4 by its deubiquitinating activity.

### 2.5. Loss of USP3 Affected Self-Renewal of hESCs

**Figure 5:**
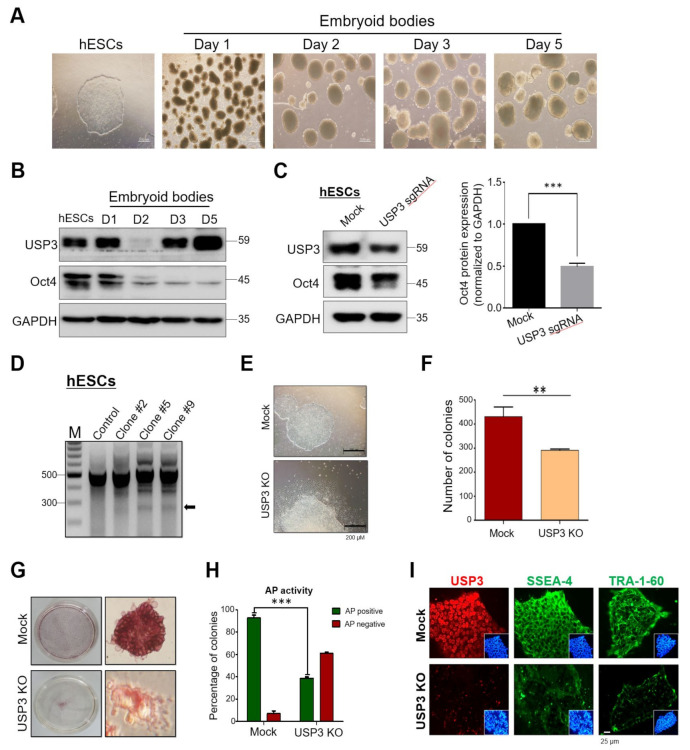
Loss of USP3 affected hESCs morphology and colony numbers. (**A**) hESCs were subjected to embryoid body (EB) differentiation for 5 days by dissociation of hESC clumps using collagenase IV and culturing the aggregates using Essential 6^TM^ Media. (**B**) Western blot analysis of differentiating EBs (days 1–5) was performed using the indicated antibodies. D1-D5 indicate the number of days of EB culture, and cells were collected at the indicated time points for Western blot analysis (**C**) hESCs were transfected with plasmids encoding *USP3* targeting sgRNA and Cas9. The expression of USP3 and Oct4 was analyzed from the pooled transfected cell population by Western blot. The band intensity of Oct4 proteins were estimated using ImageJ software and graphically represented after normalization with GAPDH. Data were presented as the means ± SDs of three independent experiments (*n* = 3). Statistical analysis was conducted by Student’s *t*-test (** *p <* 0.01). (**D**) hESCs transfected with plasmids encoding *USP3* targeting sgRNA and Cas9 were reseeded into 96-well plates and cultured for 20 days. Single round colonies were marked and analyzed by T7E1 assay. Arrowheads indicate the expected positions of DNA bands cleaved by T7E1. Mock-transfected hESCs served as control. Only T7E1-positive USP3 KO hES clones were loaded on the gel (clones #2, #5, and #9). (**E**) The morphology of mock hESCs and T7E1-positive USP3 KO hESCs (hESCs USP3 KO) under bright field microscopy. Representative image of three different USP3 KO clones. (**F**) The graph represents average number of colonies obtained from mock hESCs and USP3 KO clones. Data are represented as the mean and standard deviation of three different USP3KO clones. Statistical analysis was performed using unpaired two-tailed Student’s *t*-test: ** *p* < 0.01. (**G**) Alkaline phosphatase (AP) staining of mock hESCs and USP3 KO clones. The image is a representative image of three independent experiments. (**H**) The graphical representation of the mean percentage of AP-positive and AP-negative colonies for mock hESCs and USP3 KO clones. Statistical analysis was performed using unpaired two-tailed Student’s *t*-test: *** *p* < 0.001; *n* = 3. (**I**) Immunofluorescence analysis of mock hESCs and USP3 KO clones for the expression of USP3, Tra-1-60, and SSEA-4. Representative image of three different USP3KO clones. Inset images represent DAPI (blue)-stained nucleus.

Next, to investigate the role of USP3 during differentiation, we generated embryoid bodies (EBs) using hESCs (Figure 5A). Interestingly, Western blot analysis of EB lysates at various time points revealed a transient reduction in USP3 expression at day 2 and recovery from day 3 (Figure 5B). However, the expression of Oct4 gradually decreased during differentiation, consistent with a previous report [33] (Figure 5B).

Oct4 is the key transcription factor responsible for maintenance of pluripotent behavior in stem cells. Given that USP3 acts as a protein stabilizer of Oct4 and our observation of transient reduction in expression of USP3 at day 2 during stem cell differentiation, we investigated whether knockout of *USP3* in hESCs affected the pluripotent nature of hESCs.

We generated *USP3* knockouts (USP3 KO) in hESCs by performing single-cell-derived clone generation. hESCs were transfected with plasmids encoding Cas9 and *USP3*-targeting sgRNA1. Transfected hESC cell population demonstrated reduction in USP3 and Oct4 protein levels, as determined by Western blot analysis (Figure 5C). These hESCs were further subjected to single-cell dilution, followed by seeding into 96-well plates. Individual single-cell-derived clones that showed *USP3* gene disruption (clone #2, clone #5, and clone #9) were screened using the T7E1 assay (Figure 5D). Interestingly, microscopic analysis of these T7E1-positive, USP3-depleted hESCs revealed a morphological appearance of differentiated cells compared with mock hESCs (Figure 5E). Repeated attempts to generate USP3 KO hESCs resulted in similar morphological behavior tending towards differentiation of hESCs. The USP3 KO clones showed a significant reduction in the average number of colonies as compared to the mock hESCs (Figure 5F). We further validated the loss of pluripotency by staining mock-transfected and USP3 KO hESCs with alkaline phosphatase (AP), which is a marker commonly used to stain pluripotent stem cells. USP3 KO clones displayed less AP activity (Figure 5G) than the mock control and a significantly lower number of AP-positive colonies than the mock control (Figure 5H). Additionally, we performed immunostaining with stem cell biomarker Tra-1 and SSEA-4, which are normally expressed on the surface of undifferentiated hESCs. USP3 KO clones displayed relatively lower expression of Tra-1 and SSEA-4 when compared with mock hESCs (Figure 5I), suggesting that USP3 is a key factor involved in maintenance of pluripotent nature of hESCs.

## 3. Discussion

Post-translational regulatory mechanisms such as ubiquitination and deubiquitination complement the translational regulation of proteins in a timely and selective manner during differentiation [6,34,35]. Pluripotent stem cells have the properties of indefinite cell division while retaining their abilities to differentiate into various cell types and have been reported to exhibit high levels of proteasome activity [36]. Various Ub linkages and conformations of Ub chains in cells are generally accepted to generate diversity in molecular signaling. However, the exact mechanisms by which components of the proteasomal pathway regulate the core factors involved in determining pluripotency have not been elucidated.

Cell-cycle regulation by cyclins and CDKs is considered key towards maintenance of the self-renewal ability of stem cells [37]. hESCs proliferate rapidly, displaying an elongated S phase and a short G1 phase, and express high levels of cyclin-dependent kinase 1 (CDK1) and cyclin-dependent kinase 2 (CDK2) [37]. Oct4 plays an important role in maintaining cell-cycle-related genes in hESCs, and this regulation of the cell cycle has been postulated to have a major role in maintaining the pluripotent identity of stem cells [38]. Oct4 acts as a cell cycle promoter by removing G1 phase blocks and promoting S phase entry, governing pluripotency in stem cells [39]. There is a regulatory link between Oct4 and cell-cycle-regulating genes; Oct4 expression pattern was positively correlated with those of numerous cell cycle genes including *Gspt1, Ccna1, Ccnd2, Ccne1, Ccnb1, Ccnf, Ppp1r8*, and *Cdc25A*, indicating that Oct4 is a key cell cycle regulator [28]. Thus, perturbation in Oct4 protein level could significantly impact cell-cycle progression and pluripotency in stem cells.

In a previous study, we identified USP3 as an important regulator of the cell cycle via its deubiquitinating effect on Cdc25A [24]. The importance of Cdc25A in regulating the cell cycle in stem cells [25,27] led us to initiate this study to elucidate the role of USP3 in stem cells [24]. We first generated *USP3* knockout NCCIT cells using the CRISPR/Cas9 system and investigated the protein levels of important stem cell transcription factors. Loss of USP3 led to a decrease in the stability of Oct4; conversely, overexpression of USP3 resulted in a dose-dependent increase in the stability of Oct4 (Figure 2). Furthermore, we demonstrated that USP3 and Oct4 interact regardless of whether Oct4 is endogenously or exogenously expressed and co-localize in the nuclei of hESCs (Figure 3).

Importantly, USP3 did not show a regulator effect on mRNA level of *Oct4* (Figure 4), indicating that USP3 regulates Oct4 only at the post-translational level. We demonstrated a significant reduction in polyubiquitination of Oct4 in the presence of USP3 (Figure 4). As a functional consequence of deubiquitination of Oct4 by USP3, loss of USP3 resulted in loss of the pluripotent morphology of hESCs (Figure 5). Our findings highlight the importance of USP3 in regulating Oct4 protein level in human embryonic carcinoma cells and human embryonic stem cells. Thus, the interaction between USP3 and Oct4 is critical for maintenance of the pluripotency of hESCs.

## 4. Materials and Methods

### 4.1. Plasmids

Mammalian expression vectors encoding Flag-USP3 (cat. no. #22582, Watertown, MA, USA), HA-ubiquitin (cat. no. #18712, Watertown, MA, USA), and Cas9-2A-GFP (cat. no. #44719, Watertown, MA, USA) were purchased from Addgene (Watertown, MA, USA). To generate the catalytic mutant of USP3, we replaced the active cysteine residue at position 168 with serine by site-directed mutagenesis to produce USP3C168S (USP3CS). Cas9-2A-mRFP-2A-PAC was purchased from Toolgen (Seoul, Korea).

### 4.2. Antibodies and Reagents

The following antibodies were used: anti-USP3 (ab101473, 1:1000, abcam, Cambridge, MA, USA; GTX128238, 1:1000, Genetex, Irvine, CA, USA; and sc-135597, 1:1000, Santa Cruz Biotechnology, Dallas, TX, USA); anti-Oct4 (ab18976, 1:1000, Abcam, Cambridge, MA, USA; and sc-5279, 1:1000, Santa Cruz Biotechnology, Dallas, TX, USA); anti-Nanog (cat. no. #3580, 1:1000, Cell Signaling Technology, Danvers, MA, USA); anti-Flag (anti-DDDDK-tag) (M185-3L, 1:1000, MBL International, Woburn, MA, USA).

Anti-Lin-28 (sc-374460, 1:1000, Santa Cruz Biotechnology, Dallas, TX, USA), anti-GAPDH (sc-32233, 1:5000, Santa Cruz Biotechnology, Dallas, TX, USA), anti-Myc (sc-40, 1:1000, Santa Cruz Biotechnology, Dallas, TX, USA), anti-ubiquitin (sc-8017, 1:1000, Santa Cruz Biotechnology, Dallas, TX, USA), anti-HA (sc-7392, 1:1000, Santa Cruz Biotechnology, Dallas, TX, USA), normal mouse IgG (sc-2025, Santa Cruz Biotechnology, Dallas, TX, USA), and Protein A/G Plus Agarose beads (sc-2003, Santa Cruz Biotechnology, Dallas, TX, USA) were purchased from Santa Cruz Biotechnology (Dallas, TX, USA). TUBE 2 (cat. no. #UM402, Life Sensors, Malvern, PA, USA), MG132 (cat. no. #S2619, Selleckchem, Houston, TX, USA), and cycloheximide (CHX, cat. no. #C4859, Sigma-Aldrich, St. Louis, MO, USA) were also used. Normal rabbit IgG (cat. no. #12-370, Merck Millipore, Burlington, MA, USA), anti-Tra-1-60 (MAB4360, Merck Millipore, Burlington, MA, USA), and anti-SSEA4 (cat. no. #90231, Merck Millipore, Burlington, MA, USA) were purchased from Merck Millipore (Burlington, MA, USA).

### 4.3. Cell Culture and Treatments

CHA15 human embryonic stem cells (hESCs) and human-induced pluripotent stem cells (hiPSC-NT4-S1), established by CHA University, Seoul, South Korea, were cultured under feeder-free conditions. CHA-15 were cultured on Matrigel (cat. no. #356231, Corning Life Sciences, Tewksbury, MA, USA)-coated 35 mm dishes in mTeSR1 (cat. no. #85850, STEMCELL Technologies, Vancouver, BC, Canada), while hiPSCs were cultured on Vitronectin (cat. no. #07180, STEMCELL Technologies, Vancouver, BC, Canada)-coated 35 mm dishes in TeSR-E8 (cat. no. #05990, STEMCELL Technologies, Vancouver, BC, Canada), and subcultured every 4–5 days using Gentle Cell Dissociation Reagent (cat. no. #07174, STEMCELL Technologies, Vancouver, BC, Canada). Cells were seeded onto 35 mm dishes in mTeSR1 or TeSR-E8 supplemented with 10 μM Y-27632 (ROCK inhibitor, cat. no. #1254, Tocris Bioscience, BS, United Kingdom) to enhance cell survival and attachment.

Human pluripotent embryonal carcinoma (NCCIT) cells were cultured in RPMI media (RPMI 1640, GIBCO BRL, Rockville, MD, USA). Human embryonic kidney (HEK293) cells were cultured in DMEM media (GIBCO BRL, Rockville, MD, USA). Both RPMI and DMEM media were supplemented with 10% fetal bovine serum (GIBCO BRL, Rockville, MD, USA) and 1% penicillin and streptomycin (GIBCO BRL, Rockville, MD, USA), and cells were grown at 37 °C in a humidified atmosphere with 5% CO_2_.

### 4.4. Embryoid Body (EB) Differentiation

hESCs were differentiated in vitro to EBs. Briefly, undifferentiated colonies were detached by treatment with collagenase IV (GIBCO BRL, Rockville, MD, USA) and incubated as floating aggregates for 5 days in ultra-low attachment 6-well plates with Essential 6^TM^ Media (GIBCO BRL, Rockville, MD, USA).

### 4.5. Cas9 and sgRNA Constructs

We used plasmids encoding Cas9-2A-mRFP-2A-PAC (puromycin N-acetyl-transferase, puromycin resistance gene) and single-guide RNAs (sgRNAs) purchased from Toolgen (Seoul, South Korea). The sgRNA target sequences were designed on the basis of bioinformatics tools (www.broadinstitute.org) and cloned into the vectors as described previously [40]. Briefly, oligonucleotides containing each target sequence were synthesized (Bioneer, Seoul, South Korea), and T4 polynucleotide kinase was used to add terminal phosphates to the annealed oligonucleotides (Biorad, CA, USA). Vector was digested with BsaI and ligated the annealed oligonucleotides into the vector. Oligonucleotide sequences are listed in Table 1.

### 4.6. T7E1 Assay

Protocols for T7E1 assay were described previously [41,42]. Genomic DNA was isolated using DNeasy Blood and Tissue kit (Qiagen, Hilden, Germany) according to the manufacturer’s protocols. The region of DNA containing the nuclease target site was amplified by PCR using hemi-nested primers. PCR amplicons were denatured by heating and annealed to form DNA heteroduplex, which was then treated with 5 units of T7 E1 enzyme (New England Biolabs, MA, USA) for 20 min at 37 °C, followed by the separation of DNA fragments on 1% agarose gel by electrophoresis. Mutation frequencies were calculated using ImageJ software on the basis of band intensity. The following equation was used to calculate mutation frequency (Indel %): Indel % = 100 × (1 − [1 − fraction cleaved] 1/2), where the fraction cleaved was the total relative density of the cleavage bands divided by the sum of the relative density of cleavage and uncut bands.

Oligonucleotide primers used to amplify the PCR target for the T7E1 assay are listed in Table 1. Amplicon size of the USP3 gene and expected cleavage sizes after the T7E1 assay are summarized in Table 2.

### 4.7. Generation of DUB Knockout Single-Cell-Derived Clones

To generate USP3 knockout single-cell-derived clones, we co-transfected NCCIT or hESCs with the plasmids encoding Cas9 and sgRNA targeting USP3 or non-targeted sgRNAs (scrambled sgRNAs) at a 1:2 ratio using polyethyleneimine in NCCIT (PEI; Polysciences, Warrington, PA, USA) or Lipofectamine STEM reagent in hESCs (STEM00003, Invitrogen, Waltham, MA, USA) according to the manufacturer’s protocol. Transfected cells were sorted by FACS and reseeded into 24-well plates for recovery. Cells were dissociated and seeded again into 96-well plates to establish single-cell-derived colonies. After 15 days, single-round colonies were marked and expanded for screening by the T7E1 assay. USP3 KO-positive clones were confirmed by T7E1 and Sanger sequencing. Scrambled sgRNA-targeted cells that are T7E1-negative and showing no gene disruption of USP3 gene were used as controls for all the experiments.

T7E1-positive USP3 KO hESC clones #2, #5, and #9 generated by single-cell dilution were used for further characterization experiments described in Figure 5D–I.

### 4.8. Immunoprecipitation

For immunoprecipitation experiments, cells were lysed with RIPA buffer (cat. no. RC2002-050-00, Biosesang, Gyeonggi-do, South Korea) containing 50 mM Tris (pH 7.6), 150 mM NaCl, 2 mM EDTA, 1% Triton X-100, 0.1% SDS, 1% sodium deoxycholate, and 1 mM PMSF, followed by preclearing with Protein A/G Plus Agarose beads. Lysates were immunoprecipitated with the indicated antibodies at 4 °C overnight, followed by incubation with 20μL of Protein A/G Plus Agarose beads at 4 °C for 4 h. Immunoprecipitates were washed with lysis buffer containing 50 mM Tris (pH 7.6), 150 mM NaCl, 2 mM EDTA, 1% Triton X-100, 0.1% SDS, and 1% sodium deoxycholate, and eluted with 2X SDS sample loading buffer (cat. no. S3401, Sigma-Aldrich, St. Louis, Missouri, USA) containing 4% SDS, 20% glycerol, 10% 2-mercaptoethanol, 0.004% bromophenol blue, and 0.125 M Tris-HCl (pH 6.8). Co-immunoprecipitated proteins were denatured for 5 min at 95 °C, resolved by SDS-PAGE electrophoresis, and analyzed by immunoblotting.

### 4.9. Deubiquitination Assay

Cells transfected with various combinations of plasmids were lysed with RIPA buffer, followed by preclearing with Protein A/G Plus agarose beads as described above. Cell lysates were immunoprecipitated with anti-Oct4, anti-Myc antibodies, or TUBE agarose beads, as indicated. The immunoprecipitated proteins were eluted with 2X SDS buffer, denatured, and analyzed by Western blot with indicated antibodies.

### 4.10. Immunofluorescence

For immunofluorescence assays, NCCIT or hESCs were cultured in 4-well cell culture dishes in their respective media for 36 h. Cells were fixed with 4% paraformaldehyde (cat. no. #163-20145, Wako, Richmond, VA, USA) for 15 min and washed twice with PBS, then permeabilized with 0.25% Triton X-100 (cat. no. #0694, Amresco, Solon, OH, USA) for 10 min and blocked with 1% BSA (cat. no. #A9418, Sigma-Aldrich, St. Louis, MO, USA) in PBS for 1 h. Cells were stained with anti-USP3 and anti-OCT3/4 antibodies and incubated overnight at 4 °C. After washing with PBS, cells were incubated with Alexa Fluor-488- or Alexa Fluor-594-conjugated secondary antibodies for 1 h in the dark. DAPI was used to stain the nucleus of cells. Laser scanning confocal microscopy (TCS SP5, Leica, Wetzlar, Germany) was used to visualize fluorescence localization in cells.

### 4.11. qRT-PCR

Cells were harvested, and total RNA was isolated using TRIzol reagent (cat. no. #FATRR001, Favorgen, Ping-Tung, Taiwan). Then, 300ng of total RNA was reverse-transcribed into cDNA using Oligo dT primers (cat. no. #SO132, Thermo Scientific, Waltham, MA, USA) and Superscript III Reverse Transcriptase (cat. no. #18080-044, Invitrogen, Carlsbad, CA, USA). qRT-PCR amplification was performed using the SensiFAST SYBR No-ROX kit (cat. no. #BIO-98005, Bioline, GB, London) on a real-time PCR system (C1000 Thermal Cycler, Bio-rad, Hercules, CA, USA). GAPDH was used as the endogenous control gene. Primer information is provided in Table 3.

### 4.12. Statistics

All results are expressed as mean ± standard deviation. The significance of differences between groups was assessed using Student’s *t*-test. All statistical analyses were performed using GraphPad Prism 5 software (GraphPad Software, Inc. San Diego, CA, USA). Differences were considered statistically significant at *p* < 0.05.

## Figures and Tables

**Table 1:** Oligonucleotides used in this study.

Gene	sgRNA	Direction		Sequence (5′ to 3′)
Oligonucleotides used for sgRNA plasmid construction				
*USP3*	sgRNA1	FP		GACTCAGCCAAGTTCCCCAA
		RP		TTGGGGAACTTGGCTGAGTC
	sgRNA2	FP		AGTTCAGCACACAGTATGTA
		RP		TACATACTGTGTGCTGAACT
Oligonucleotides used for amplify the PCR target for the T7E1 assay				
*USP3*	sgRNA1	I PCR	FP	TCGGAGTTACACGTTCTACGG
			RP	CTGCGGAGAAGCGCGG
		II PCR	FP	TCGGAGTTACACGTTCTACGG
			RP	GCCTCGGGAAACAAAGGA
	sgRNA2	I PCR	FP	CTTGCCTGAGCCTACTCTTGT
			RP	AGGATGGATGAGGAACGGGA
		II PCR	FP	TCACTACATGATTGACTGCTGTT
			RP	AGGATGGATGAGGAACGGGA

**Table 2:** PCR amplicon and T7E1 cleavage sizes.

Gene	sgRNA	PCR Size	Cleavage Size	Orientation
*USP3*	sgRNA1	507	280 + 227	Sense
	sgRNA2	504	274 + 230	Sense

**Table 3:** Oligonucleotides used for qRT-PCR.

Gene	Direction	Sequence (5′ to 3′)
*Oct4*	FP	GCTGGATGTCAGGGCTCTTT
	RP	TCAAGAGATTTATCGAGCACCTTCT
*GAPDH*	FP	GTCATCCCTGAGCTGAACGG
	RP	CCACCTGGTGCTCAGTGTAG

## Data Availability

All data generated or analyzed during this study are included in this published article and its Supplementary Materials Files.

## Supplementary Materials

The following are available online at https://www.mdpi.com/article/10.3390/ijms22115584/s1.
