# Supplementary material for: Ubiquitin-Specific Protease 3 Deubiquitinates and Stabilizes Oct4 Protein in Human Embryonic Stem Cells
Source: Int J Mol Sci. 2021 May 25;22(11):5584. doi: 10.3390/ijms22115584 (PMC8197518; doi:10.3390/ijms22115584)
Supplement: Supplementary file 1 [file ijms-22-05584-s001.zip › ijms-1235323-supplementary.pdf]

Supplementary Figures

Ubiquitin-specific protease 3 deubiquitinates and stabilizes Oct4 protein in human embryonic stem cells

Byung Ho Rhie[1], Ainsley Mike Antao[1], Janardhan Keshav Karapurkar[1], Minseong Kim[1], Won Jun Jo[1], Suresh Ramakrishna[1,2]*, and Kye-Seong Kim[1,2]*

[1]Graduate School of Biomedical Science and Engineering, Hanyang University, Seoul 04763, South Korea

[2]College of Medicine, Hanyang University, Seoul 04763, South Korea

Running title: USP3 regulates Oct4 protein turnover in stem cells

***Corresponding authors**

SR (E-mail: suri28@hanyang.ac.kr, suresh.ramakris@gmail.com);

KS (E-mail: ks66kim@hanyang.ac.kr)

Table of Contents

Figure S1. Deubiquitination of Myc-Oct4 by USP3.

Figure S2. The original uncropped images for immunoblots in the main figures.

Figure S3. Triplicate images of western blots for significance values.

Supplementary Figure S1. Deubiquitination of Myc-Oct4 by USP3.

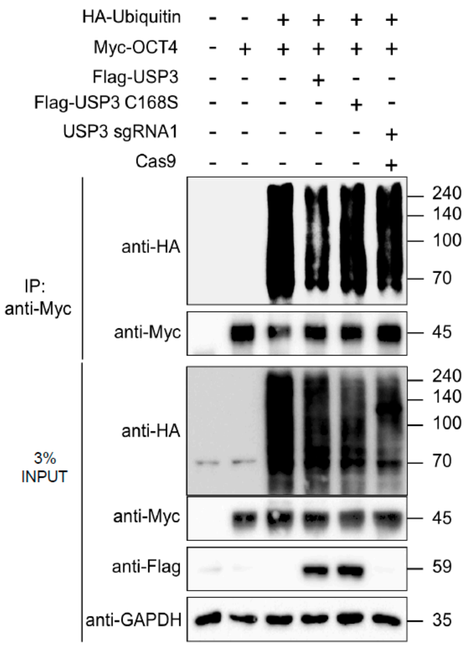

Supplementary Figure S1: Deubiquitinating effect of USP3 on exogenous Oct4 was analyzed by transfecting Myc-Oct4 and HA-Ubiquitin along with Flag-USP3, Flag-USP3CS, or sgRNA targeting USP3 into HEK293 cells. Oct4 deubiquitination was confirmed by IP with anti-Myc antibody and immunoblotted with anti-HA antibody.

Supplementary Fig. S2: The original uncropped images for immunoblots in the main figures.

Supplementary Figure S1

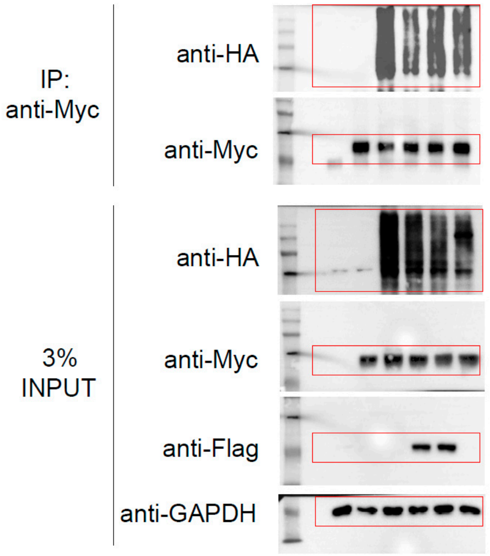

Figure 1E

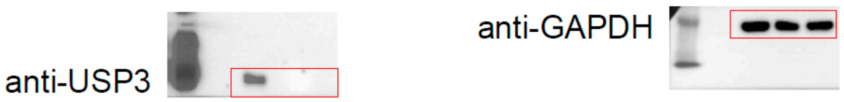

Figure 2A

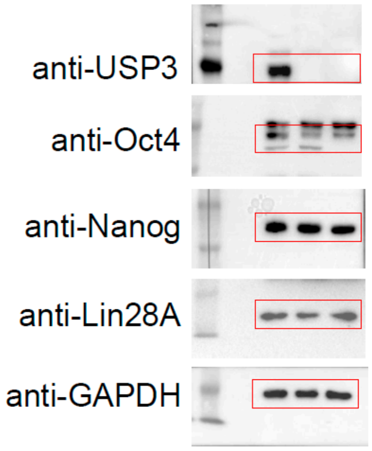

Figure 2B

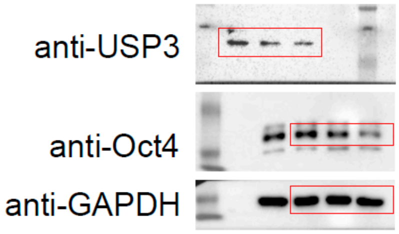

Figure 2C

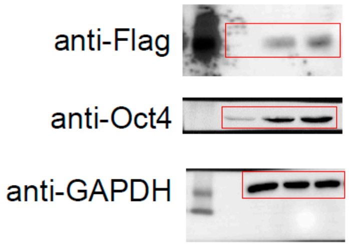

Figure 2D

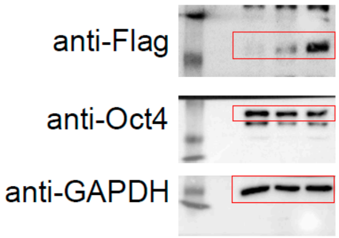

Figure 2E

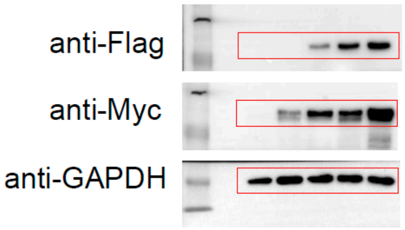

Figure 2F

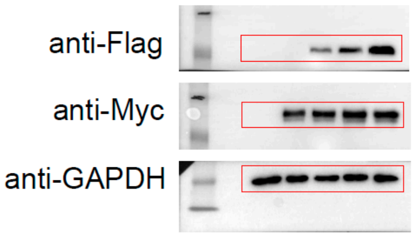

Figure 2G

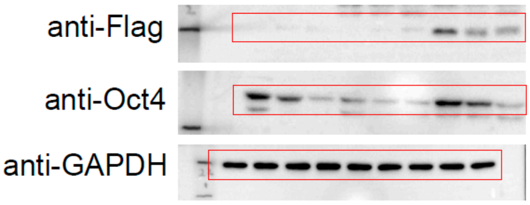

Figure 2H

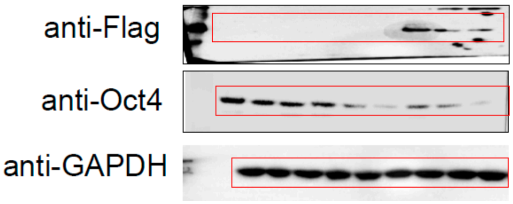

Figure 3A

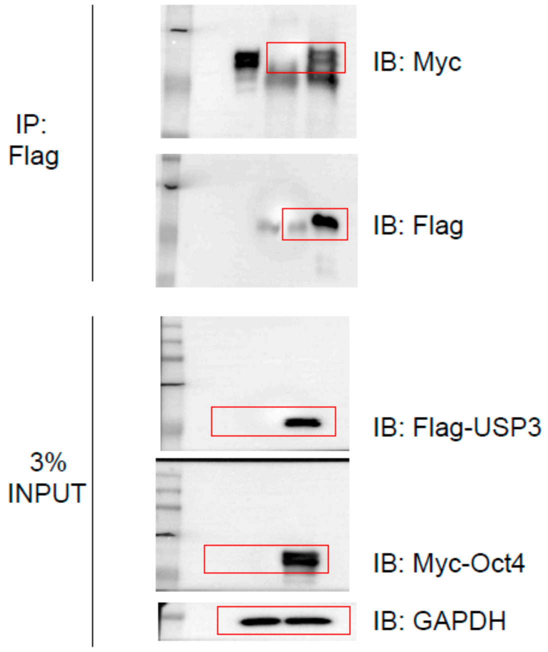

Figure 3B

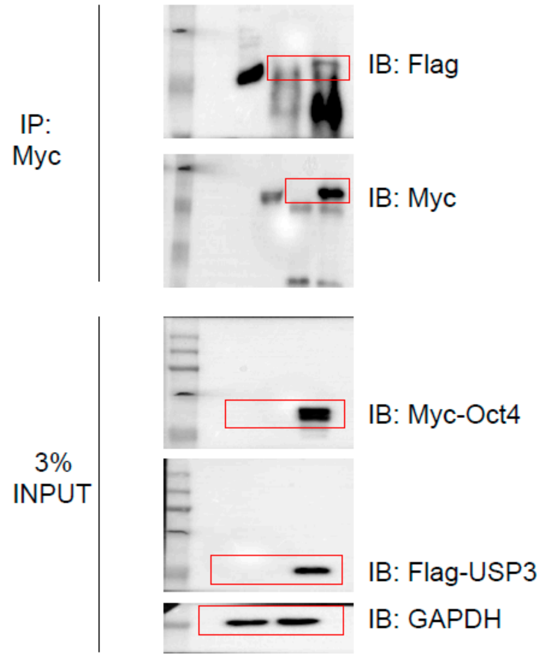

Figure 3C

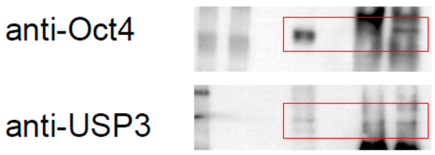

Figure 3D

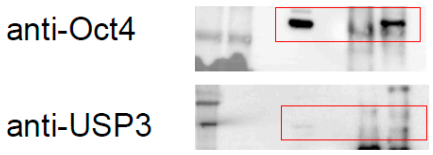

Figure 4B

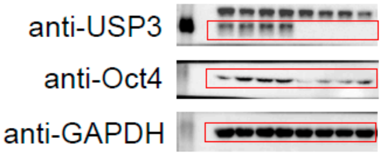

Figure 4C

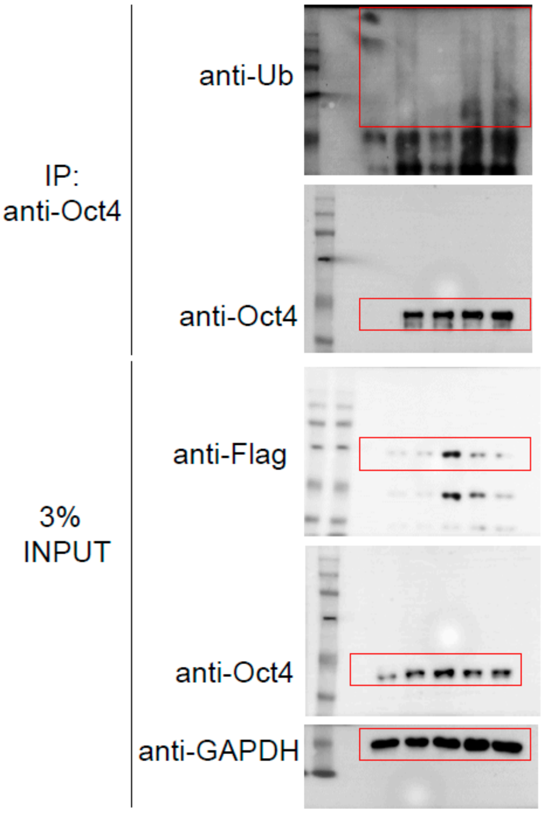

Figure 4D

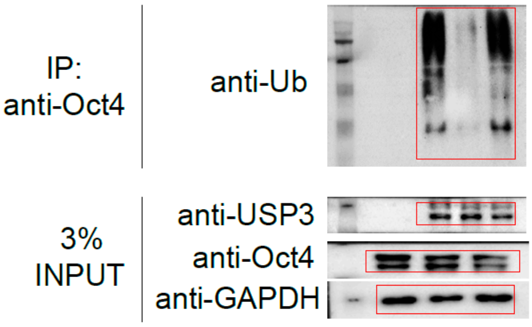

Figure 4E

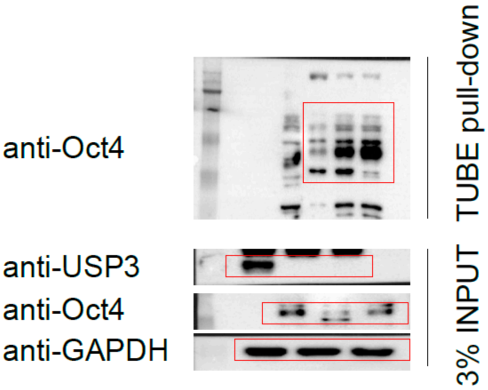

Figure 5B

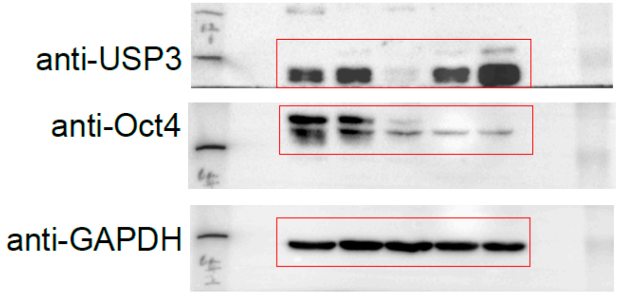

Figure 5C

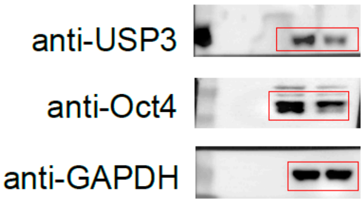

Figure S3. Triplicate images of western blots for significance values.

Figure 2B

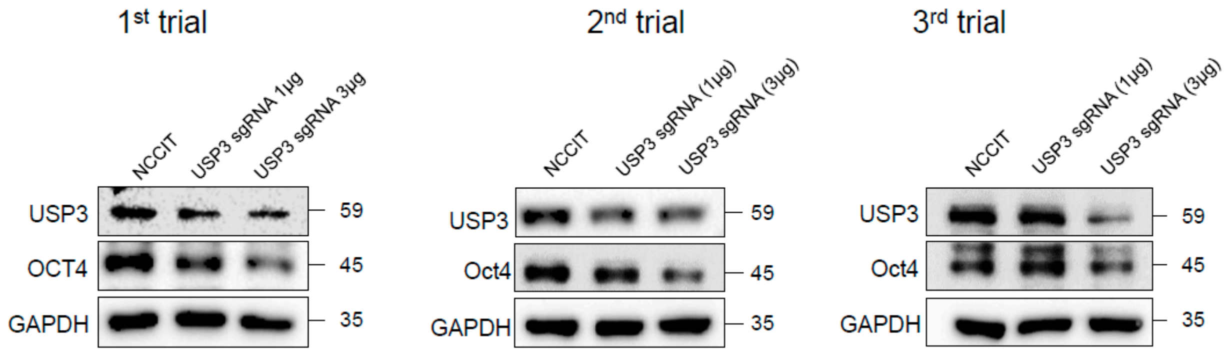

Figure 2C

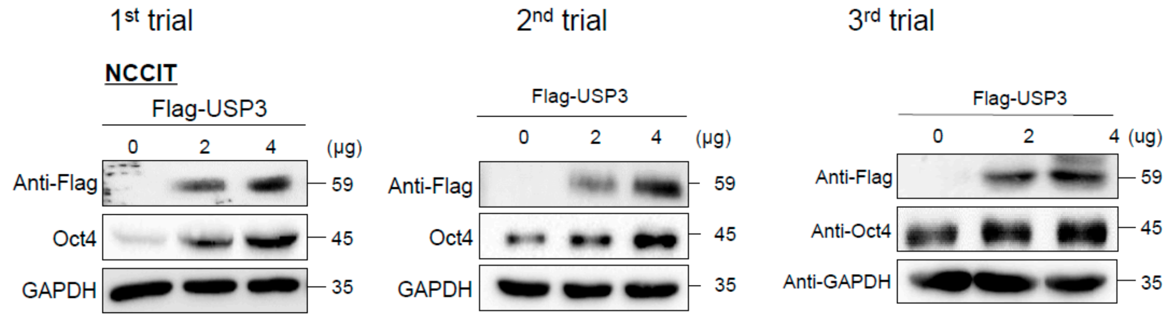

Figure 2D

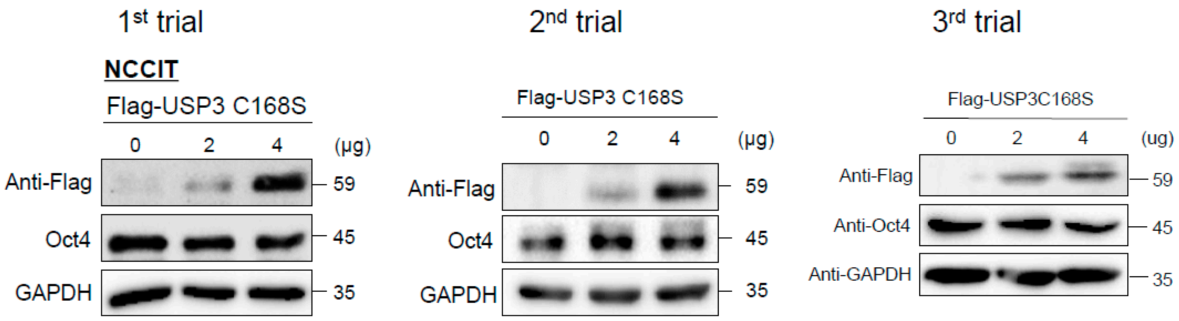

Figure 5C

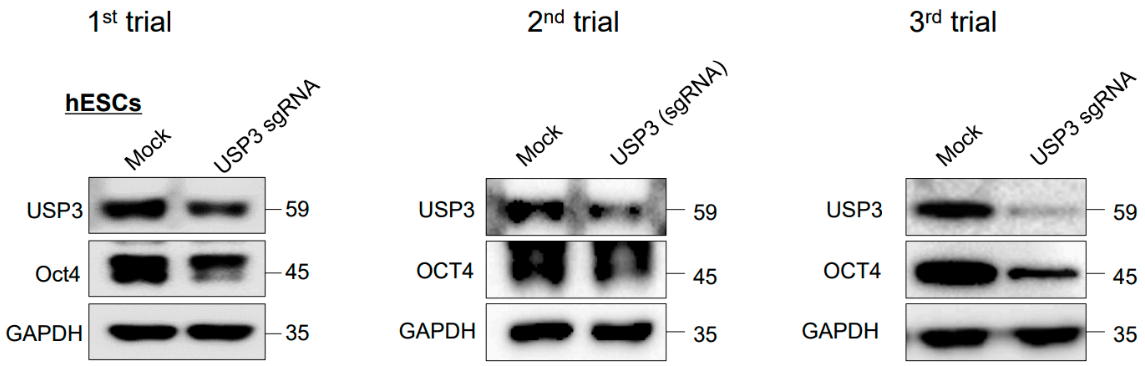
1st trial
2nd trial
3rd trial
hESCs
Mock
USP3 sgRNA
Mock
USP3 (sgRNA)
Mock
USP3 sgRNA
USP3
Oct4
GAPDH
59
45
35
USP3
OCT4
GAPDH
59
45
35
USP3
OCT4
GAPDH
59
45
35
